# The Gut–Lung Microbiome Crosstalk and Pulmonary Disease

**DOI:** 10.3390/biom16060833

**Published:** 2026-06-04

**Authors:** Diren Beyoğlu, Jeffrey R. Idle

**Affiliations:** College of Pharmacy and Health Sciences, Western New England University, Springfield, MA 01119, USA

**Keywords:** gut–lung axis, gut microbiome, lung microbiome, dysbiosis, short-chain fatty acids, amino acid metabolites, dietary fuels

## Abstract

Both the gut and the lungs possess a microbiome, a community of commensal bacteria, archaea, fungi, and viruses that perform important housekeeping functions in those organs. The colonic microbiome primarily ferments indigestible dietary fibers into essential short-chain fatty acids, synthesizes essential vitamins, regulates the mucosal immune system, and forms a protective barrier against pathogenic colonization. The lung microbiome maintains respiratory health primarily by regulating mucosal immunity, providing a physical barrier against invading pathogens, and producing beneficial metabolites. Several colonic microbiota metabolites, including the short-chain fatty acids acetate, propionate, and butyrate, together with the tryptophan metabolites indole-3-acetate and indole-3-propionate, secondary bile acids, and the polyamines spermidine and putrescine, are transported to the lungs via the gut–lung axis. These colonic microbiota biomolecules suppress lung inflammation, strengthen immune homeostasis, and reduce the severity of respiratory diseases. In contrast, lung microorganisms and their metabolites can travel to the gut via the gut–lung axis, influencing intestinal immune responses and potentially leading to an imbalance of gut microorganisms or dysbiosis. This means that respiratory diseases may lead to digestive issues, intestinal inflammation and chronic diseases. Here, we have reviewed this crosstalk and its impact on the principal pulmonary diseases: asthma, chronic obstructive pulmonary disease, cystic fibrosis, bronchogenic carcinoma, COVID-19, interstitial lung diseases, pneumonia, and tuberculosis. It is concluded that the gut microbiome plays a significant part in lung health and disease. Diet, tobacco smoking and electronic cigarette vaping all impact both the gut and lung microbiomes.

## 1. Introduction

That human fecal excrement might contain living organisms was first proposed in 1681 by Antoni van Leeuwenhoek. He examined his own feces using a homemade single-lens microscope and observed tiny motile organisms, which he called “animalcules” (small animals) [1,2]. He also observed his animalcules in scrapings of his own unbrushed teeth, his nose, and in water to which had been added peppercorns [3,4]. These were perhaps both the discovery of bacteria and the first evidence for an intestinal microbiota [2]. The Human Microbiome Project Consortium analyzed 4788 specimens from 242 screened and phenotyped adults. Men were sampled at 15 body habitats and women at 18 [5]. Two methodologies were employed to determine the taxa present in the stool samples produced by each of the 242 subjects. The first was 16S rRNA gene amplicon analysis, and the second was whole genome shotgun analysis. The former we have previously discussed in detail [6] and is specific for bacteria and archaea only. The latter can characterize bacteria, archaea, fungi, viruses, and protists, such as algae and amoebae. While 16S rRNA gene sequencing is targeted and can resolve down to the genus level, shotgun metagenomics has a higher resolution and can yield information regarding species and even strain [7].

The purpose of this review is to describe the gut and lung microbiomes, then illuminate the crosstalk between them, emphasizing the important roles that the gut microbiota play in the pathogenesis and potential therapies of common pulmonary diseases.

## 2. The Gut Microbiome

Five phyla mainly comprise the organisms of the human intestinal microbiota, although these proportions vary from study to study [6]. In one report [8], the proportions were Bacillota (formerly Firmicutes) (79%—*Ruminococcus*, *Clostridium*, *Eubacteria*), Bacteroidota (formerly Bacterioidetes) (17%—*Porphyromonas*, *Prevotella*), Pseudomonadota (formerly Proteobacteria) (1%), Actinomycetota (formerly Actinobacteria) (*Bifidobacterium*) (2.5%) and Verrucomicrobiota (0.1%). Another estimate used 9 million unique gut bacterial genes [9] from 13 bacterial phyla, with Bacillota and Bacteroidota comprising 64.9% and 30.4%, respectively, with the remaining 11 phyla occupying 4.7% (Pseudomonadota, 1.6%; Verrucomicrobiota, 1.5%; Actinomycetota, 1.4%; Others, 0.2%). These estimates were all based upon the analysis of feces. The human gut has an estimated surface area of 200 m^2^, the same size as a tennis court, and this provides a major surface to be colonized by the microbiota [10]. Accordingly, biopsies of the gastrointestinal mucosa were taken by endoscopy from a single healthy human subject (female, age 54) at the following sites: proximal jejunum, distal ileum, ascending colon, and rectum. 16S rRNA sequencing was made of a total of 347 clone libraries from the mucosal biopsies. The six bacterial phyla Bacillota, Bacteroidota, Pseudomonadota, Actinomycetota, Verrucomicrobiota, and Fusobacteriota (formerly Fusobacteria) were quantitated [11]. The jejunum had the least bacterial diversity and the ascending colon the greatest. *Streptococcus* sequences dominated the jejunum samples, while the distal ileum, ascending colon and rectum samples were all dominated by Bacteroidota, together with *Clostridium* clusters XIVa and IV [11]. It has been estimated that the human gut microbiome consists of at least 1800 genera and ~15,000–36,000 species of bacteria [12]. Older estimates put the number of species at 500–1000 [13].

The microbiota pattern is highly variable between individuals, and the following conditions strongly influence the bacterial community: diet, infection, medication, genetics, and age [14]. There is a gradient of bacteria across the gastrointestinal tract (GIT), from 10^1^ cells/g in the stomach, 10^3^ cells/g in the duodenum, 10^4^ cells/g in the jejunum, 10^7^ cells/g in the ileum, and 10^12^ cells/g in the colon [10]. One might reasonably ask, “Where do these bacteria come from?” Colonization of the human gut begins immediately after birth but differs considerably between vaginal delivery and C-section delivery. In the former case, the neonate develops a microbiota similar to that of the mother’s vagina [15], including bacteria such as *Limosilactobacillus reuteri* and *L. rhamnosus*. In the latter case, the neonatal microbiota comprises bacteria found in hospitals and on the mother’s skin. These infants lack early colonization of *Bacteroides* spp. and *Bifidobacterium* spp., showing higher levels of opportunistic pathogens like *Enterococcus* spp. and *Klebsiella* spp. [16]. *Bacteroides* spp. do not appear until 6 to 18 months of age. There was also evidence for mother-to-child transmission of rectal rather than vaginal bacteria [17].

The principal functions of the gut microbiota are manifold: (i) digestion of dietary fibers and complex carbohydrates that the host is unable to break down, (ii) generation of the short-chain fatty acids (SCFAs) acetate, propionate, butyrate, valerate, lactate, isobutyrate and isovalerate, which provide energy to the host and reduce inflammation, (iii) synthesis of vitamins K, B_1_, B_2_, B_3_, B_5_, B_6_, B_7_, B_9_, and B_12_ [18], (iv) strengthening of the intestinal barrier to protect against pathogens, and (v) regulation of the immune system [19]. Perturbation of this symbiosis leads to a condition known as dysbiosis, an imbalance in the microbial communities (bacteria, archaea, fungi, viruses) living in the gut, where beneficial microbes decrease while harmful ones increase [20]. This disruption compromises the immune system and digestive health, often causing symptoms like bloating, constipation, diarrhea, and fatigue, and is linked to chronic diseases [21].

We have discussed in detail the phenomenon of atopobiotic bacteria, especially with respect to the urinary, blood, and hepatic microbiomes [22]. These organisms have been described as viable but not culturable (VBNC) bacteria [23].

## 3. The Lung Microbiome

The healthy lung was traditionally considered sterile, but this notion has been challenged by emerging molecular approaches that enable comprehensive examination of microbial communities [24]. The first characterization of the microbial communities inhabiting the airways was reported in 2010 [25]. The bronchial tree was not sterile but rather contained an average of 2000 bacterial genomes per cm^2^ surface sampled. Pathogenic Pseudomonadota, in particular *Haemophilus* spp., were much more frequent in the bronchi of adult asthmatics or patients with COPD than in controls. Children with asthma had elevated Pseudomonadota and diminished Bacteroidota, in particular, *Prevotella* spp. This pulmonary microbiome was clearly perturbed in asthma [25]. In contrast to other organ systems, the respiratory tract harbored a homogenous microbiota that decreased in biomass from the upper to the lower respiratory tract. One investigator stated that the healthy lung does not contain a consistent, distinct microbiome but instead contains low levels of bacterial sequences largely indistinguishable from upper respiratory flora [24]. However, this viewpoint is not universally shared. Others report that the lung microbiota of healthy individuals mainly comprises the genera *Streptococcus* (Bacillota), *Fusobacterium* (Fusobacteriota), *Haemophilus* (Pseudomonadota), *Bacteroides* (Bacteroidota), *Pseudomonas* (Pseudomonadota), *Prevotella* (Bacteroidota), and *Neisseria* (Pseudomonadota) [26].

### The Origin of the Lung Microbiome

It has been stated, “The principles of respiratory microbiology are being re-evaluated and re-written, starting with the debunked myth of lung sterility” [27]. The main contributors to the lung microbiome are oropharyngeal bacteria (such as *Prevotella*, *Veillonella*, and *Streptococcus*). Other sources include inhalation of environmental microbes (air contains 10–1000 bacterial cells per liter [28]) and potentially the gut microbiome, which influences pulmonary immunity. Microaspiration of oral secretions from oropharyngeal and nasopharyngeal source communities, which occurs even in healthy individuals (particularly during sleep), is the primary driver of bacterial influx [29,30]. Microbial clearance from the respiratory tract is mediated by mucociliary transport, tussive mechanisms—prevalent even in healthy populations—and a tiered immunological response that transitions from immediate, non-specific innate mechanisms to highly targeted adaptive defenses [31]. The entire respiratory tract, from the nose and mouth down to the alveoli, functions as a cohesive ecosystem. Within this system, various physiological gradients and specific niches control how the microbiome moves, settles, and grows. In a healthy state, the lungs are continuously exposed to bacteria, yet it has been claimed that these microbes typically remain only temporarily rather than establishing permanent colonies [32].

Research indicates the lung microbiome significantly influences brain autoimmunity through a connection known as the lung–brain axis [33,34] and may be involved in the pathogenesis of multiple sclerosis [34,35]. In all other regards, the lung microbiome plays a part in various lung diseases [36], as will be discussed below.

## 4. Gut–Lung Microbiome Crosstalk

The gut–lung axis is a highly coordinated, bidirectional communication network driven by gut microbiota-originated metabolites such as SCFAs, tryptophan metabolites, and polyamines. The regulation of these metabolites involves upstream mechanisms (production, processing, and barrier transport within the gut) and downstream mechanisms (systemic transport, immune cell reprogramming, and targeted pulmonary signaling) [37].

The gastrointestinal and respiratory systems share a common embryological origin, the endodermal primitive gut tube that forms during the first three weeks of embryogenesis [38]. As essential components of the common mucosal immune system (CMIS), both the lungs and intestines function as expansive, high-surface-area barriers designed to defend against environmental pathogens through innate and adaptive immunity. Mounting evidence suggests a bidirectional relationship, the lung-gut axis, wherein, for example, intestinal disturbances influence respiratory infections, and respiratory diseases lead to gastrointestinal complications [39,40]. As stated above, the gut microbiota produces copious amounts of SCFAs, and these can reach the lungs via the circulation. SCFAs, primarily acetate, propionate, and butyrate produced by the gut microbiota, operate as key mediators in the gut–lung axis to suppress lung inflammation, strengthen immune homeostasis, and reduce the severity of respiratory diseases. They reduce airway inflammation, inhibit harmful histone deacetylase activity, and can even induce lung tumor cell death [41,42,43,44]. The SCFAs enter the bloodstream and bind to G-protein coupled receptors (like GPR41/GPR43) on immune cells. In the bone marrow, SCFAs alter hematopoiesis, shifting dendritic cell precursors toward a phenotype that limits Th2-mediated allergic responses in the lungs [45,46].

It is axiomatic that a high intake of dietary fiber leads to a greater production of SCFAs by the gut microbiota and therefore a higher potential delivery of SCFAs to the lung. The initial premise was investigated in a comparison of the fecal bacteria of European children with children from rural Burkina Faso, whose diet is exceptionally high in fiber [47]. It was reported that significant differences were found in gut microbiota between the two groups. Burkina Faso children showed a significant enrichment in Bacteroidota (Bacteroidetes) and depletion in Bacillota (Firmicutes), with an exclusive abundance of bacteria from the genera *Prevotella* and *Xylanibacter*, known to contain a collection of bacterial genes for cellulose and xylan hydrolysis, which were completely absent in the European children [47]. Moreover, the African children had increased levels of the SCFAs propionate and butyrate, which were at least four times higher than those of the European children. These features were linked to the low occurrence of allergies and autoimmune diseases in this Burkina Faso rural population [47].

SCFAs are not the only gut microbiota metabolites that may influence lung health. Evidence is emerging that secondary bile acids, which are produced from primary bile acids by the gut microbiota [6], may also modify pulmonary processes. Specifically, isolithocholic acid was found to be the most prominent immunomodulator in the lung. The findings establish microbiota-derived bile acids as systemic immunometabolic modulators [48]. These observations are in contradistinction to an earlier study concluding that bile acid signaling was a leading trigger for the development of chronic respiratory disease [49]. Additionally, tryptophan metabolites produced by gut microbiota, such as indole-3-propionic acid and indole-3-acetic acid, function as anti-inflammatory signals that protect the lungs by activating the aryl hydrocarbon receptor. These metabolites reduce excessive inflammation and mitigate acute lung injury and allergic airway hyperreactivity by modulating immune cells such as macrophages and T-cells [50,51,52,53].

A pertinent question is what happens to the lungs during gut microbiota dysbiosis. Gut microbiota dysbiosis disrupts the gut–lung axis, leading to increased pulmonary inflammation and reduced immune tolerance. Reduced production of protective SCFAs by gut microbes allows pro-inflammatory signals to travel via the bloodstream, increasing airway hyperresponsiveness and lung tissue damage [54,55,56].

In summary, SCFAs, in particular propionate and butyrate, together with certain secondary bile acids and tryptophan metabolites, all produced by the gut microbiota, have beneficial effects on pulmonary health via the gut–lung axis. The upstream processing and downstream signaling of the major groups of gut microbiota metabolites are shown in Table 1.

**Figure 1:**
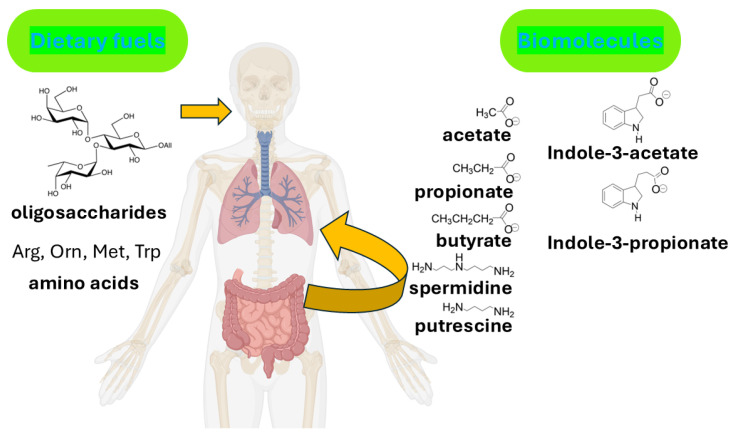
The colonic microbiota generation of biomolecules from dietary fuels and transport to the lungs by the gut–lung axis.

By corollary, what effect does perturbation of the lung microbiota have on the gut? Lung microorganisms and their metabolites can travel to the gut via the bloodstream, influencing intestinal immune responses and potentially leading to gut dysbiosis. This means that respiratory diseases may lead to intestinal inflammation. As a primary cause of global morbidity and mortality, respiratory infections of bacterial and viral origin represent a significant public health challenge. Over the last decade, research has increasingly highlighted the role of the lung microbiota in pulmonary disease; however, the specific pathways through which these microbial communities affect the intestinal environment remain to be fully elucidated [62]. The current understanding of the immune crosstalk between the respiratory and gastrointestinal tracts focuses on the mechanisms of immune cell recruitment and migration during respiratory infections [63]. In summary, the immune crosstalk between the respiratory and gastrointestinal tracts is a bidirectional communication network part of the CMIS. It facilitates the migration of immune cells and signals, allowing gut microbiota metabolites to regulate lung immunity and enabling respiratory infections to alter gut microbiota composition. The gut–lung axis and the impact of dietary fuels on the generation of biomolecules by the colonic microbiota is shown in Figure 1.

## 5. Gut–Lung Microbiome Crosstalk and Pulmonary Disease

The gut–lung axis significantly influences various respiratory conditions. Gut microbiota dysbiosis directly affects immune responses, altering disease severity in asthma, chronic obstructive pulmonary disease (COPD), cystic fibrosis (CF), and lung infections such as pneumonia and COVID-19 [64]. Gut microbiota metabolites, particularly SCFAs, tryptophan catabolites, and secondary bile acids, regulate pulmonary immunity through G-protein-coupled receptors, histone deacetylase inhibition, and aryl hydrocarbon receptor signaling [65]. The principal fungi in the gut microbiome are *Candida*, *Aspergillus* and *Wallemia*, comprising about 0.1% of the microbiota and commonly referred to as the mycobiome [66]. Recent research is now revealing how, in addition to gut bacteria, gut fungi may influence lung immunity in conditions such as asthma, lung cancer, and various respiratory infections [66].

### 5.1. Asthma

Asthma affects an estimated 260–360 million people globally, making it a major noncommunicable disease and the most common chronic disease among children. While age-standardized rates have declined since 1990, the absolute burden is rising due to population growth, with ~436,000 deaths in 2021. Principal causes of this heterogeneous disease include genetics, environmental allergens, air pollution, smoking, and rising obesity rates [67,68,69,70]. The gut–lung axis modulates immune responses in the lungs, significantly impacting asthma development and severity. A balanced gut microbiome produces metabolites that reduce airway inflammation (see above), whereas gut dysbiosis, an imbalance from poor diet or antibiotics, promotes Th2 inflammation, increasing asthma risk [71]. This occurs by dysbiotic bacteria altering microbial metabolism, often increasing toxic metabolites like trimethylamine *N*-oxide (TMAO), which drives CD4+ T-cell differentiation toward a Th2 phenotype, resulting in elevated immunoglobulin E (IgE) and chronic inflammation [72]. Dysbiosis is linked to a wide range of conditions both inside and outside the digestive tract. These include inflammatory bowel disease, infections, food allergies, asthma, diabetes, obesity, multiple sclerosis, autism, gum disease, and colorectal cancer [73]. Many of these are recognized as comorbidities that can exacerbate asthma, make it harder to control, or trigger severe attacks. These conditions often act through shared systemic inflammation, immune system dysregulation [74]. In essence, the many conditions that provoke gut dysbiosis can aggravate asthma via the gut–lung axis.

Intestinal fungi are among the most powerful drivers of the T-helper 2 (Th2) immune responses that dominate allergic asthma. Fungi within the gut microbiome appear to have an effect on asthma development. Fungal dysbiosis in the gut microbiome is associated with bacterial dysbiosis and later asthma development [75,76]. Moreover, an overgrowth of specific gut fungi (such as *Candida* or *Rhodotorula*) during infancy strongly correlates with a high risk of developing childhood asthma.

The gut virome, predominantly comprising bacteriophages, acts as an upstream controller of asthma by constantly shaping the gut’s bacterial landscape and antiviral readiness [77]. By regulating gut bacterial populations, specific phages influence the production of protective metabolites (like SCFAs) and modulate the immune system’s maturation, thereby reducing or heightening asthma and allergy risk. According to Barnett et al. (2019), “The role of the virome in the gut microbiome and its effect on asthma remains to be discovered” [78].

### 5.2. Chronic Obstructive Pulmonary Disease (COPD)

Chronic obstructive pulmonary disease (COPD) is the fourth leading cause of death worldwide, causing 3.5 million deaths in 2021, approximately 5% of all global deaths. Tobacco smoking accounts for over 70% of COPD cases in high-income countries. In low- and middle-income countries, tobacco smoking accounts for 30–40% of COPD cases, with household air pollution a major risk factor [79]. COPD is driven by inflammation, leading to permanent lung damage through conditions such as chronic bronchitis and emphysema. Recent research points to high levels of IL-1-like cytokines in the lungs of COPD patients, suggesting that a protein complex called the inflammasome plays a key role. Most studies have specifically focused on the NLRP3 (NLR family pyrin domain containing protein 3) inflammasome, a signaling hub that activates the enzyme caspase-1 to produce and release the inflammatory cytokines, typically IL-1β and IL-18 [80].

Substantial evidence confirms that patients with COPD are frequently deficient in antioxidants, vitamins, and fiber micronutrients required to maintain eubiosis of the gut microbiota. The unhealthy Western-style diet is associated with an increased risk of COPD and an accelerated decline of pulmonary function. Intake of fruit, vegetables, dietary fiber, vitamins C, D, and E, polyphenols, such as curcumin, and β-carotene were individually associated with lower COPD risk, whereas consumption of processed meat was associated with higher COPD risk [81,82,83,84].

Recently, a prospective, multicenter, longitudinal and controlled study of 60 stable COPD patients and 30 controls was reported. The authors conducted 16S rRNA sequencing in oropharyngeal swabs, sputum, bronchoalveolar lavage fluid and stool. The gut and airway microbiomes were highly dissimilar both in patients and controls. In stable patients with COPD, both throughout the airways and in stool, co-existing bacterial taxa were associated with pertinent clinical traits (such as airflow limitation severity and exacerbations). Further research was recommended on the role of microbiome dysbiosis in COPD pathogenesis [85].

While fungi comprise a small fraction of the gut microbiome, distinct shifts in their composition correlate closely with COPD severity [86]. In severe COPD, the mycobiome rewires microbial networks, fostering a strong, harmful interaction between *Candida* and pathogenic bacteria [86]. Imbalances in gut fungi can promote the release of pro-inflammatory cytokines into the bloodstream, exacerbating chronic airway inflammation [66].

Studies show a significantly lower richness and diversity of the gut virome in patients with COPD compared to healthy individuals [87]. Viral populations responsible for regulating pathogenic bacteria decline in COPD, leading to weakened viral-bacterial interactions and decreased bacterial susceptibility [87]. Specific viral species (such as *Clostridium* phage and *Myoviridae*) have been shown to correlate positively with pulmonary ventilation functions, suggesting that the virome could act as a future diagnostic biomarker for COPD [87].

### 5.3. Cystic Fibrosis

Cystic fibrosis (CF) is the most common inherited autosomal recessive disease in the Caucasian population. The disease affects multiple organ systems and can have a wide variety of clinical presentations. More than 2000 mutations of the recessive *CFTR* (cystic fibrosis transmembrane conductance regulator) gene can cause cystic fibrosis. Different mutations affect how much CFTR protein the cells make and how well the protein works. With the most common gene mutation, part of the CFTR gene is missing. The mutation makes a CFTR protein that cannot stay in the correct shape. Some *CFTR* mutations cause cells to make hardly any CFTR protein at all. Normally, the CFTR protein controls how salt and water move in and out of cells. In people who have CF, the faulty CFTR protein causes mucus to become thick and sticky because it contains less water. In addition, the sweat glands make extra-salty sweat [88]. A total of 162,428 (144,606–186,620) people are estimated to be living with CF across 94 countries [89].

In the gastrointestinal (GI) tract, CFTR dysfunction results in low intestinal pH due to decreased bicarbonate secretion, thick and congealed mucus, a lack of endogenous pancreatic enzymes, and reduced gut motility. These abnormalities, combined with antibiotic therapies, drive GI inflammation and generate dysbiosis [90,91]. A microbial imbalance develops, characterized by a higher amount of pro-inflammatory microbiota, such as *Escherichia* and *Enterococcus*, than immunomodulatory genera, such as *Bacteroides* and *Bifidobacterium*. The abundance of Enterobacteriaceae that comprises over 30 genera and more than 100 species, particularly *Escherichia coli*, has been shown to be 10-times higher in CF compared to healthy controls [92].

The gut microbiome can have a modulating effect on immune function. For example, *Bacteroides fragilis* modulates Th1/Th2 balance, and segmented filamentous bacteria directly stimulate Th17 cell differentiation, whereas *Clostridium* spp. induce Treg production. Moreover, metabolites such as SCFAs are involved in promoting recruitment, as well as maturation of immune cells, which provide protection against an inflammatory response [92]. In CF, therefore, there are direct effects of the genetic defect on the lungs and gut, together with other organs. In addition, these effects may be amplified through the gut–lung axis.

In CF, gut dysbiosis driven by CFTR dysfunction alters the mycobiome and virome, leading to reduced microbial diversity. This imbalance exacerbates intestinal inflammation, aggravates malabsorption, and negatively impacts distal organs via the gut–lung axis, worsening respiratory health and pulmonary exacerbations [93]. Fungal overgrowth constantly stimulates pattern recognition receptors (e.g., Dectin-1) on host immune cells. This provokes a chronic, low-grade systemic inflammatory response, worsening the malnutrition and GI issues often seen in CF [94].

### 5.4. Bronchogenic Carcinoma

Primary lung cancer is the leading cause of cancer cases and deaths worldwide, with an estimated 2.5 million new cases and 1.8 million deaths in 2022. More than 1.3 million cases in men and nearly 500,000 lung cancer cases in women are preventable, with the majority attributable to tobacco smoking (60–70%), followed by air pollution and occupational exposure [95]. Non-small-cell lung cancer (NSCLC) accounts for over 85% of cases [96]. The example of lung cancer is atypical, with three microbiomes to be considered, those residing in the airways, in the gut, and in the tumor itself [97].

In the gut microbiome, the production of SCFAs such as butyrate can influence lung tumors via the gut–lung axis by reducing inflammation in the tumor microenvironment, which inhibits tumor progression [98]. Cross-sectional studies reported that fecal diminution of butyrate producers, such as *Faecalibacterium prausnitzii* or enhancement of *Fusobacterium* spp., was linked to NSCLC [99]. The commonest polyamines produced by the gut microbiota are putrescene and spermidine (see Figure 1), which are synthesized from arginine, methionine, and ornithine by a large number of bacterial species, especially those of the *Bacteroides* genus [100]. Polyamines can inhibit immune cell function in the lungs, and this can also inhibit tumor progression [101]. Accordingly, lesser abundance of *Bacteroides* and *Faecalibacterium* was associated with higher risk of NSCLC, mediated by reduced CD8+ T-cell infiltration [102], that allowed tumor progression [103]. Therefore, the gut microbiome can either inhibit or enhance lung cancer development.

The lung microbiome comprises mainly Bacteroidota (Bacteroidetes), Bacillota (Firmicutes), Pseudomonadota (Proteobacteria), and Actinomycetota (Actinobacteria). Several lung microbiome genera have been investigated in relation to the initiation and progression of bronchogenic carcinoma. These included *Pseudomonas*, *Streptococcus*, *Prevotella*, *Fusobacterium*, *Veillonella*, *Porphyromonas*, *Haemophilus*, *Acinetobacter*, *Acidovorax*, which have been reported as having a marked presence, together with several other bacterial genera, in lung cancer patients. While these bacteria may promote the growth of lung cancers, others, for example *Neisseria*, may retard lung tumor growth [104]. Therefore, the lung microbiome may also inhibit or enhance lung cancer development.

Lung tumors contain a unique and distinct microbiome, a community of bacteria, fungi, and viruses that is different from healthy lung tissue. These microorganisms reside inside cancer and immune cells, shaping the tumor microenvironment to promote tumor progression and affect immune response. The tumor microenvironment represents a multifaceted ecosystem characterized by the cohabitation of malignant cells with a heterogeneous population of immune infiltrates, including macrophages, granulocytes, mast cells, NK cells, dendritic cells, and lymphocytes, as well as stromal and endothelial components. Intricate intercellular signaling within this niche modulates a dynamic equilibrium that can either facilitate oncogenic progression and metastasis or exert tumor-suppressive effects [105]. While this area of research is in its infancy, it promises to significantly advance our comprehension of oncogenesis and tumor progression.

Gut fungi interact with immune cells (such as dendritic cells and macrophages) via pattern-recognition receptors like Dectin-1. This can either promote a healthy, tumor-suppressing immune response or create an immunosuppressive environment that fosters tumor growth [106].

Specific components of the virome can trigger robust immune responses, though viral dysbiosis can accelerate chronic inflammation, which is a known driver of lung cancer initiation [107].

### 5.5. Coronvirus Disease COVID-19

Coronavirus disease (COVID-19) is an infectious respiratory disease caused by the severe acute respiratory syndrome coronavirus 2 (SARS-CoV-2) virus, which emerged in late 2019. COVID-19 resulted in over 6 million deaths worldwide in less than four years. The virus spreads primarily through airborne particles when an infected person breathes, talks, or coughs, often causing symptoms like fever, cough, fatigue, and loss of taste or smell 2–14 days after exposure. While many experience mild illness, it can cause severe pneumonia, organ failure, or death [108]. The cumulative number of global reported COVID-19 cases was, at the time of writing, 779,201,796, 103 million of which were in the United States of America [109].

The gut microbiome significantly influences COVID-19 severity and recovery by regulating immune responses via the gut–lung axis. COVID-19 causes gut dysbiosis, a reduction in beneficial bacteria (e.g., *Faecalibacterium prausnitzii*) and an increase in opportunistic pathogens, which correlates with higher disease severity, systemic inflammation, and persistent “Long COVID” symptoms. The protein that mediates SARS-CoV-2 host cell entry is ACE2, which is highly expressed on the membrane of gastrointestinal cells. Subsequently, infection can lead to gut microbiota dysbiosis, which is associated with inflammation and other systemic infections and diseases [110]. Importantly, pathobionts can escape the gut into the systemic circulation during dysbiosis, potentially leading to dangerous secondary infections during COVID-19 [111].

The lung microbiome plays a critical role in COVID-19 severity by regulating local immune responses; dysbiosis often correlates with higher disease severity. COVID-19 infection alters the lung microbial composition, reducing beneficial bacteria and increasing opportunistic pathogens (like *Streptococcus* and *Staphylococcus*) associated with poorer clinical outcomes and longer ICU stays. Changes in the lung microbiota often involve an increase in oral opportunistic pathogens (like *Capnocytophaga* and *Veillonella*) in the lungs, likely facilitated by infection-related changes or mechanical ventilation [112]. Reduced levels of beneficial, SCFA-producing bacteria in the lung microbiome were associated with heightened inflammation and worse outcomes [113].

The gut mycobiome and virome undergo severe, persistent dysbiosis during COVID-19. This imbalance, marked by an overgrowth of fungal pathogens and altered viral populations, impairs gut barrier integrity and drives systemic inflammation, exacerbating disease severity [114]. Unlike healthy subjects, the gut mycobiome in COVID-19 patients is unstable and can remain altered even after the viral infection is cleared from the respiratory tract [114,115].

COVID-19 infection is linked to a prolonged imbalance in the gut virome, including alterations in both bacteriophages and eukaryotic viruses [114]. An imbalanced virome can impair the gut–lung axis where gut microbes modulate systemic T-cell and interferon responses. This disruption compromises the host’s antiviral immunity and contributes to hyper-inflammatory states [116,117].

### 5.6. Interstitial Lung Disease

Interstitial lung disease (ILD) is an umbrella term for over 200 chronic respiratory conditions causing progressive inflammation and irreversible scarring (fibrosis) of lung tissue, which stiffens lungs and hinders oxygen absorption. Primary symptoms include persistent dry cough, shortness of breath, and fatigue. While often caused by environmental exposures or autoimmune diseases, the most aggressive form, idiopathic pulmonary fibrosis, has no known cause and a poor prognosis if untreated [118]. The global prevalence of ILD is approximately 58 per 100,000 people based on 2019 Global Burden of Disease data, with estimated ranges varying from 6.3 to 71 per 100,000 individuals. Prevalence increases with age and is generally higher in males, with cases more than doubling between 1990 and 2019 due to population aging [119].

Gut microbiome dysbiosis drives systemic inflammation and worsens lung fibrosis. Studies show that reduced microbial diversity and an increase in pro-inflammatory bacteria in the gut correlate with higher disease severity and poorer outcomes in ILD patients [120]. Regarding the lung microbiome, in idiopathic pulmonary fibrosis, 16S rRNA sequencing of bronchoalveolar lavage fluid revealed increased bacterial burdens in lungs, enriched for pathogenic genera like *Haemophilus*, *Streptococcus*, *Neisseria*, and *Veillonella* spp. [121].

Fungal species such as *Candida* and *Aspergillus* in the gut are closely monitored by the immune system. If these fungal communities overgrow, they release antigens that can provoke and sustain aberrant Th17 immune responses, which accelerate lung tissue scarring [66].

The gut virome is essential for training the host’s innate immune defense. However, viral dysbiosis in the gut can compromise this balance. Furthermore, systemic viral infections can travel to and severely exacerbate existing lung diseases, often triggering acute respiratory deteriorations in patients with idiopathic pulmonary fibrosis or other fibrotic ILDs [122].

### 5.7. Pneumonia

Pneumonia is an infection that inflames the alveoli in one or both lungs, causing them to fill with fluid or pus. It is caused by bacteria, viruses, or fungi, leading to symptoms like cough, fever, chills, and breathing difficulties. It can range from mild to life-threatening, particularly in older adults, children, and those with weakened immune systems [123]. Pneumonia is a leading infectious cause of death globally, causing approximately 2.5 million deaths in 2023. It heavily impacts vulnerable populations, with over 600,000 deaths in children under five annually. Pneumonia is most prevalent in South Asia and sub-Saharan Africa, representing a major global health burden. Major pathogens causing pneumonia deaths in children under five include *Streptococcus pneumoniae* (197,000), *Klebsiella pneumoniae* (75,000), *Pseudomonas aeruginosa* (46,000), *E. coli* (33,000), *Staphylococcus aureus* (31,000), *Respiratory syncytial virus (RSV)* (27,000), and *Influenza virus* (25,000) [124].

Both the gut and lung microbiomes significantly affect pneumonia susceptibility, severity, and recovery. A healthy lung microbiome helps protect against pathogens, while dysbiosis can increase susceptibility to infection, drive inflammation, and correlate with worse outcomes in pneumonia patients [125].

The gut mycobiome and virome interact with the gut–lung axis, profoundly impacting pneumonia severity and host resistance. Through immune-modulating metabolites and cross-kingdom interactions, these communities influence whether the lungs can effectively clear respiratory infections [66,126]. Commensal gut fungi act as a low-level “vaccine.” They stimulate the immune system to produce circulating anti-fungal antibodies, which prime alveolar macrophages and help protect the lungs from systemic fungal infections. Similarly, balanced gut phages help regulate overall mucosal immunity [127].

Severe pneumonia, particularly in the ICU, alters both the lung and gut virome. An influx of specific bacteriophages in the respiratory tract and compromised intestinal barriers can lead to a convergence that precedes secondary hospital-acquired pneumonia [62,126].

### 5.8. Tuberculosis (TB)

Tuberculosis (TB) is a contagious infection caused by *Mycobacterium tuberculosis* bacteria, primarily attacking the lungs. Spread through air via coughs or sneezes, it exists as active TB disease (symptomatic) or latent TB infection (inactive). It is curable with antibiotics but can be fatal if untreated. About a quarter of the global population is estimated to have been infected with TB bacteria. In general, people with TB infection do not feel sick and are not contagious. About 5–10% of people infected with TB will eventually get symptoms and develop TB disease. Babies and children are at higher risk of developing the disease if they are infected. TB is usually treated with antibiotics and can be fatal without treatment. In certain countries, the Bacille Calmette-Guérin (BCG) vaccine is given to babies or small children to prevent TB. The vaccine prevents deaths from TB and protects children from serious forms of TB [128].

The human microbiome, particularly in the gut and lungs, plays a crucial role in TB susceptibility, progression, and treatment. TB infection and treatment often cause significant dysbiosis, while a healthy microbiota helps modulate the immune response against *Mycobacterium tuberculosis* [129,130]. It is believed that the crosstalk between the host immune system and gut and lung microbiomes could provide new insights into TB pathogenesis [131].

Active TB is associated with an increased proportion of certain fungal species and a decreased fungal-to-bacterial ratio. Opportunistic pathogens often expand in the gut, leading to prevalent gastrointestinal symptoms like bloating and nausea [132].

During TB, alterations in the phageome directly reduce beneficial commensal bacteria [133]. Because antituberculosis therapy profoundly disrupts the microbial community, individuals recovering from TB may have compromised gut microbiomes, which correlates with an increased chance of TB recrudescence or re-infection [134,135].

### 5.9. Summary

Acute and chronic, infectious and noninfectious, pulmonary diseases are affected by both the gut and lung microbiomes [136,137,138]. This also includes the mycobiome and the virome, as we have discussed. Although the organisms residing in the lungs are of immediate interest with respect to lung diseases, paradoxically, the intestinal microbiota may be of even greater importance in a wide range of respiratory diseases, as we have discussed above.

## 6. Effect of Diet, Smoking and Vaping on the Gut and Lung Microbiomes

Diet is a well-studied variable with respect to the gut microbiome. We have reviewed this subject extensively in relation to longevity [139,140,141,142]. A healthy diet acts as the primary fuel source for the gut microbiota, directly shaping its composition, diversity, and function. Consuming high-fiber, plant-based foods (fruits, vegetables, whole grains) promotes a diverse, beneficial microbiome that produces health-boosting metabolites, such as SCFAs, while low-fiber, processed foods diminish diversity and cause inflammation [143,144]. This is illustrated in Figure 1.

Lifestyle factors have long been investigated with respect to pulmonary diseases. That cigarette smoking might be associated with lung cancer was first reported in 1931 by an insurance company statistician called Frederick Hoffman. His series of 27 patients with lung cancer comprised 18 (67%) who were heavy smokers [145]. In the intervening century, the first reports emerged in 1950 that claimed a causal relationship between tobacco smoking and lung cancer [146,147], culminating in the Surgeon General’s Report of 1964, which reviewed over 7000 studies over a period of 14 months and with the assistance of 150 consultants [148]. A detailed account of the landmark papers (1929–2023) has been given [149]. Despite the tens of thousands of scientific reports on tobacco smoking and lung diseases, only a handful of papers have emerged regarding the effects of tobacco smoking on the lung microbiome [150,151,152].

Smoking disrupts the gut microbiome, causing dysbiosis, which increases the risk of inflammatory diseases like Crohn’s disease. It reduces beneficial bacteria, alters SCFA production, increases fecal pH, and can increase the growth of mouth-related bacteria in the gut, such as *Streptococcus* [153]. Furthermore, *Prevotella* spp. appear significantly increased in smokers and former smokers but not in electronic cigarette users, with a progressive increase in *Desulfovibrio* with the number of pack-years of cigarettes smoked and an increase in *Alphaproteobacteria* in current versus never smokers. The authors concluded, “Maintaining the balance of intestinal microbiota represents a new possibility for therapeutic approaches to smoking-related diseases. Further research is needed in this direction” [154].

The effect of electronic cigarette use on the microbiome has been investigated. Electronic cigarettes (e-cigarettes, electronic nicotine delivery systems [ENDS]) are battery-powered devices, including vapes, pens, and pods, that heat a liquid, usually containing nicotine, propylene glycol, glycerin, and flavorings, into an inhalable aerosol. While often marketed for smoking cessation, they are not FDA-approved for this purpose. They are generally considered less toxic than conventional cigarettes [155]. E-cigarettes disrupt the human microbiome by altering bacterial composition, reducing microbial diversity, and promoting an environment that fosters inflammation and opportunistic pathogens. Vaping impacts the oral, nasal, and gut microbiomes, potentially increasing the risk of respiratory infections, periodontal disease, and gut barrier dysfunction [156,157,158]. In the oral microbiome, *Porphyromonas* and *Veillonella* spp. were significantly elevated in vapers [156], and in another study, *Veillonella* and *Haemophilus* spp. were prominent [159]. Regarding the gut microbiome, the effect of vaping constituents on the gut barrier was investigated using human and murine gut-derived organoids in culture, which made sweeping conclusions based upon these in vitro models [160].

E-cigarettes significantly alter the lung microbiome by reducing bacterial diversity and increasing the abundance of potential pathogens, leading to heightened inflammation and decreased resistance to respiratory infections. Vaping disrupts the delicate balance of microbial ecosystems in the airways, causing effects similar to tobacco smoking, including higher concentrations of pathogens like *Staphylococcus aureus* [161].

At the time of publication [162], six percent of Americans, including 3 million high school students, used e-cigarettes. The first human study on the effects of e-cigarette exposure on the oral microbiome was reported by these investigators, who compared e-cigarette users with cigarette smokers and never smokers. E-cigarette use was associated with higher levels of Gram-negative facultative bacteria, while cigarette smoking was selectively enriched for Gram-negative anaerobes. *Candidatus Saccharibacteria oral taxon TM7x* and species belonging to the genera *Abiotrophia*, *Aggregatibacter*, *Eikenella*, *Granulicatella*, *Cardiobacterium*, *Hemophilus*, *Johnsenella*, *Kingella*, *Lachoanaerobaculum*, *Lautropia*, *Leptotrichia*, *Mogibacterium*, *Ottowia*, *Parvimonas*, *Peptostreptococcus*, *Rothia*, *Rhodobacter*, *Selenomonas*, and *Veillonella* demonstrated significantly greater abundance in ENDS users when compared to both never smokers and smokers. The discovery of a distinct core microbiome in e-cigarette users, significantly different from both smokers and nonsmokers, indicated that e-cigarette aerosol exerted a unique influence on the oral microbial environment compared to conventional tobacco smoke [162]. Several investigators have established a relationship between respiratory diseases and perturbations of the oral microbiome [163,164,165,166].

In summary, the gut and lung microbiomes are both significantly affected by diet, tobacco smoking and vaping.

## 7. Microbiome-Targeted Treatments

Restoring microbial equilibrium through targeted therapies offers a highly promising new approach for lung diseases that are influenced by gut dysbiosis [37].

### 7.1. Probiotics

We have previously discussed the origin of probiotics [6], live beneficial microorganisms, such as *Bifidobacterium* and *Lactobacillus* spp., that are consumed in various dietary forms or as supplements in order to promote eubiosis in the gut microbiota [167]. By introducing beneficial bacteria, probiotics help calibrate immune response, reduce systemic inflammation, and protect against respiratory infections [168,169]. Probiotic genera like *Lactobacillus* produce SCFAs that enter the gut–lung axis, helping to lessen allergic airway inflammation in conditions like asthma and COPD [170]. Extensive research has demonstrated their potential. In particular, in mouse models, the depletion or absence of segmented filamentous bacteria leads to impaired immune responses and worsened outcomes following respiratory infections [171]. Moreover, *Lactobacillus* interventions significantly reduce pulmonary inflammation in COPD models via SCFA-driven pathways along the gut–lung axis [172]. Administration of probiotics therefore provides a potential strategy for future adjuvant therapy.

### 7.2. Fecal Microbiota Transplantation (FMT)

FMT has historically been utilized as a highly effective treatment for recurrent *Clostridioides difficile* infection. It works by transferring healthy, screened donor stool into the patient’s colon, restoring the gut’s natural microbial balance and preventing the return of *Clostridioides difficile* [173]. Recent clinical trials and animal models demonstrate that FMT can significantly suppress lung inflammation, rebalance immune cells, and restore metabolic pathways in chronic and acute respiratory conditions. FMT suppresses the inflammatory Toll-like receptor 4 (TLR4) pathway, reduces cell death in lung tissues, and repairs alveolar damage [174]. FMT is proving to be a critical tool in managing multi-drug-resistant severe pneumonia and associated lung injury. Case reports note that severe pneumonia patients given healthy donor microbiota show reduced tracheal secretions, negative sputum cultures, and cleared lung CT scans. Mechanistically, FMT suppresses dangerous opportunistic pathogens (like *Klebsiella pneumoniae*) and rebalances the regulatory Treg/Th17 balance to heal severe, infection-driven lung damage [175,176].

### 7.3. CRISPR-Cas Systems

The use of CRISPR-Cas systems to modify the gut microbiota represents a cutting-edge therapeutic paradigm for treating lung diseases via the gut–lung axis. Rather than relying on traditional, non-specific probiotics or FMT, CRISPR-Cas technology allows for the precise, sequence-specific editing of the intestinal microbiome [37,177]. CRISPR/Cas9 or Cas12a tools can be engineered into common gut commensals, such as *Bacteroides* or *Lactobacillus* spp., to upregulate or insert metabolic pathways that amplify SCFA synthesis [178]. These last authors concluded, “We envision that CRISPR/Cas-based genome editing tools for *Bacteroides* will greatly facilitate mechanistic studies of the gut commensal and the development of engineered live biotherapeutics.”

## 8. Conclusions

It is now clear that the intestinal microbiome plays a key role in managing the biochemistry and immunology of the lung. Not only does the gut microbiota exert influence over the pathogenesis of lung diseases, but it offers unique avenues for their treatment or prevention through the use of probiotics, fecal microbiota transplantation, and CRISPR-Cas technologies. These new vistas have gained increasing promise at a time when antibiotic resistance and the paucity of novel antibiotic developments are a reality.

## Figures and Tables

**Table 1:** The upstream processing and downstream signaling of the major groups of gut microbiota metabolites that enter the gut–lung axis.

Gut Microbiota Metabolite Group	Upstream Processing	Downstream Signaling	Refs.
Short-chain fatty acids (SCFAs)	**Production**: Fermentation of dietary fiber by anaerobic commensals, e.g., *Bacteroides* and *Faecalibacterium prausnitzii.* **Transport**: Passive diffusion or active transport by SLC5A8 across the gut epithelial barrier.	**Immune**: In bone marrow, SCFAs reprogram immune progenitor cells, making dendritic cells and macrophages less reactive to harmless allergens. **In lungs**: SCFAs bind to GPR41 and GPR43 on epithelial cells, suppressing allergic airway inflammation and reducing tissue damage.	[57,58]
Tryptophan metabolites (Trpms)	**Production**: Tryptophanase in, e.g., *Lactobacillus* spp. converts tryptophan into indoles. **Transport**: Passive diffusion across the gut epithelial barrier.	**Immune**: Trpms bind to AhR on immune cells. **In lungs**: Trpms bind to AhR on lung epithelial cells leading to ↑ survival of intraepithelial lymphocytes, ↑ protective IL-22, and tightening of the alveolar epithelial barrier to prevent pathogen invasion.	[59]
Polyamines	**Production**: Synthesized from Arg and Orn by *Escherichia coli* and *Bacteroides* spp. **Transport**: Actively transported across the gut epithelial barrier.	**Immune**: Block activation of NF-κB in alveolar macrophages. **In lungs**: Downregulates production of pro-inflammatory cytokines TNFα and IL-6.	[60,61]

## Data Availability

No new data were created or analyzed in this study.

## Abbreviations

The following abbreviations are used in this manuscript: SCFAs, short-chain fatty acids; COPD, chronic obstructive pulmonary disease; CMIS, common mucosal immune system; TMAO, trimethylamine *N*-oxide; CFTR, cystic fibrosis transmembrane conductance regulator; NSCLC, non-small-cell lung cancer; SARS-CoV-2, severe acute respiratory syndrome coronavirus 2; ILD, interstitial lung disease; TB, tuberculosis.
